# Supplementary material for: A high-resolution spatial map of cilia-associated proteins in the human fallopian tube
Source: Nat Commun. 2026 Apr 20;17:3616. doi: 10.1038/s41467-026-71692-6 (PMC13096173; doi:10.1038/s41467-026-71692-6)
Supplement: Supplementary file 1 — Supplementary Information [file 41467_2026_71692_MOESM1_ESM.pdf]

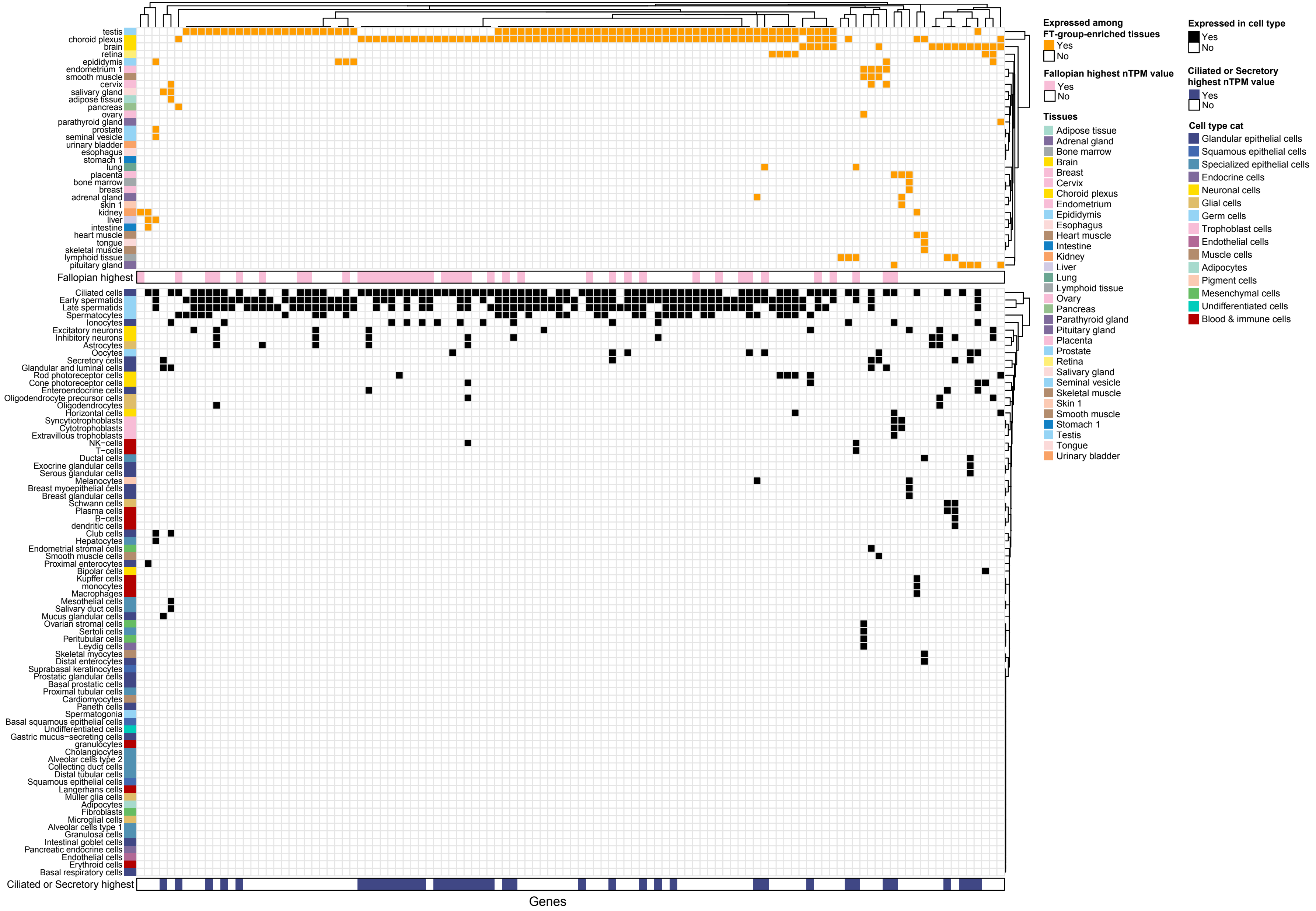

Supplementary Figure 1. Heatmap showing the binary relationship of group-enriched tissues (upper) and elevated cell type specificities (lower) for 114 group-enriched genes in FT. The upper heatmap shows which tissues are in the group "group-enriched" for each gene (columns), with a legend showing if the peak RNA level is in FT tissue or not. The lower heatmap shows which cell types are present in the cell type elevated groups for each gene with a legend showing the peak RNA level in Ciliated and/or Secretory cells or not.

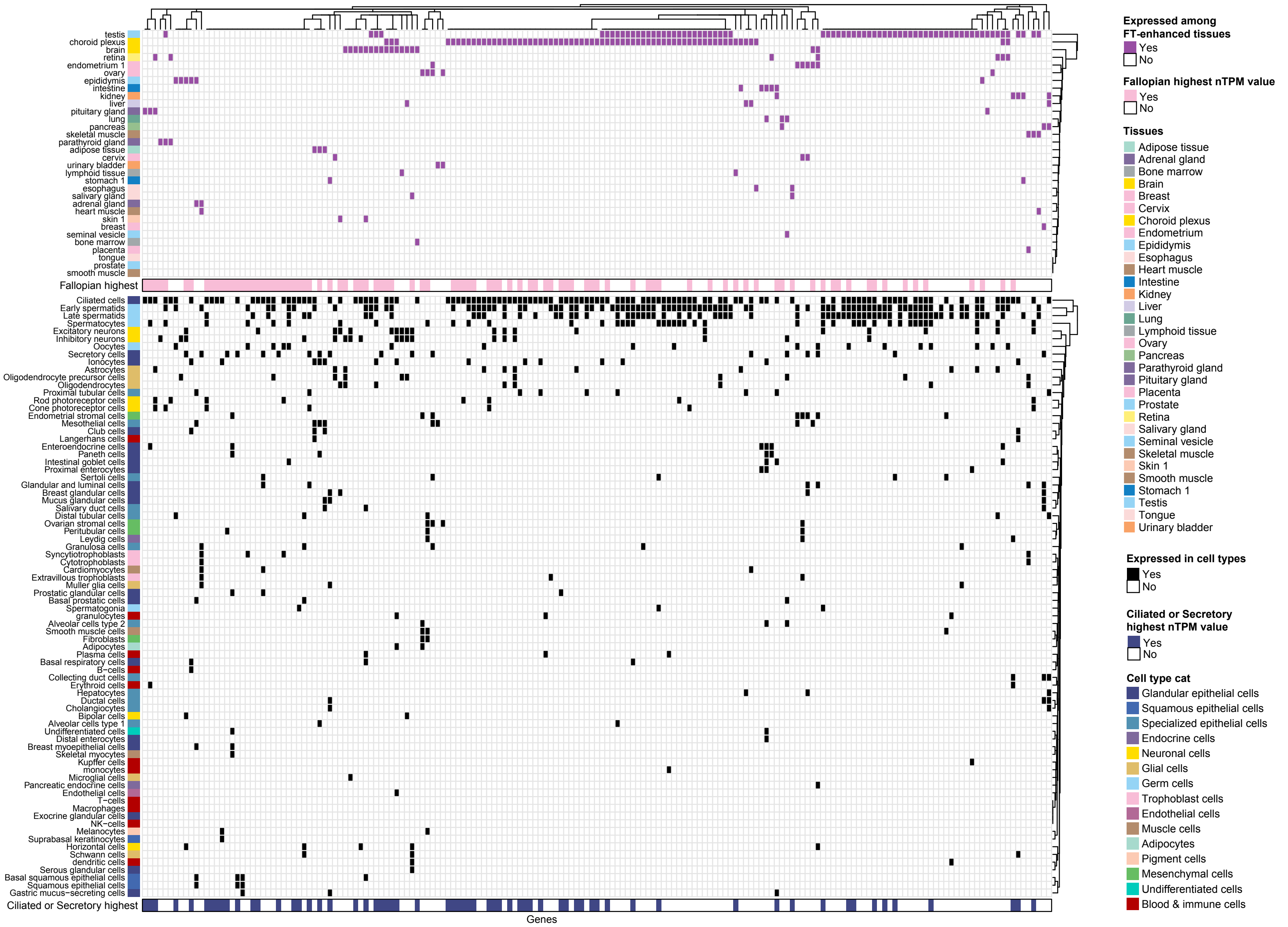

Supplementary Figure 2. Heatmap showing the binary relationship of enhanced tissues (upper) and elevated cell type specificities (lower) for 117 enhanced genes in FT. The upper heatmap shows which tissues are in the group "enhanced" for each gene (columns), with a legend showing if the peak RNA level is in FT tissue or not. The lower heatmap shows which cell types are present in the cell type elevated groups for each gene with a legend showing the peak RNA level in Ciliated and/or Secretory cells or not.

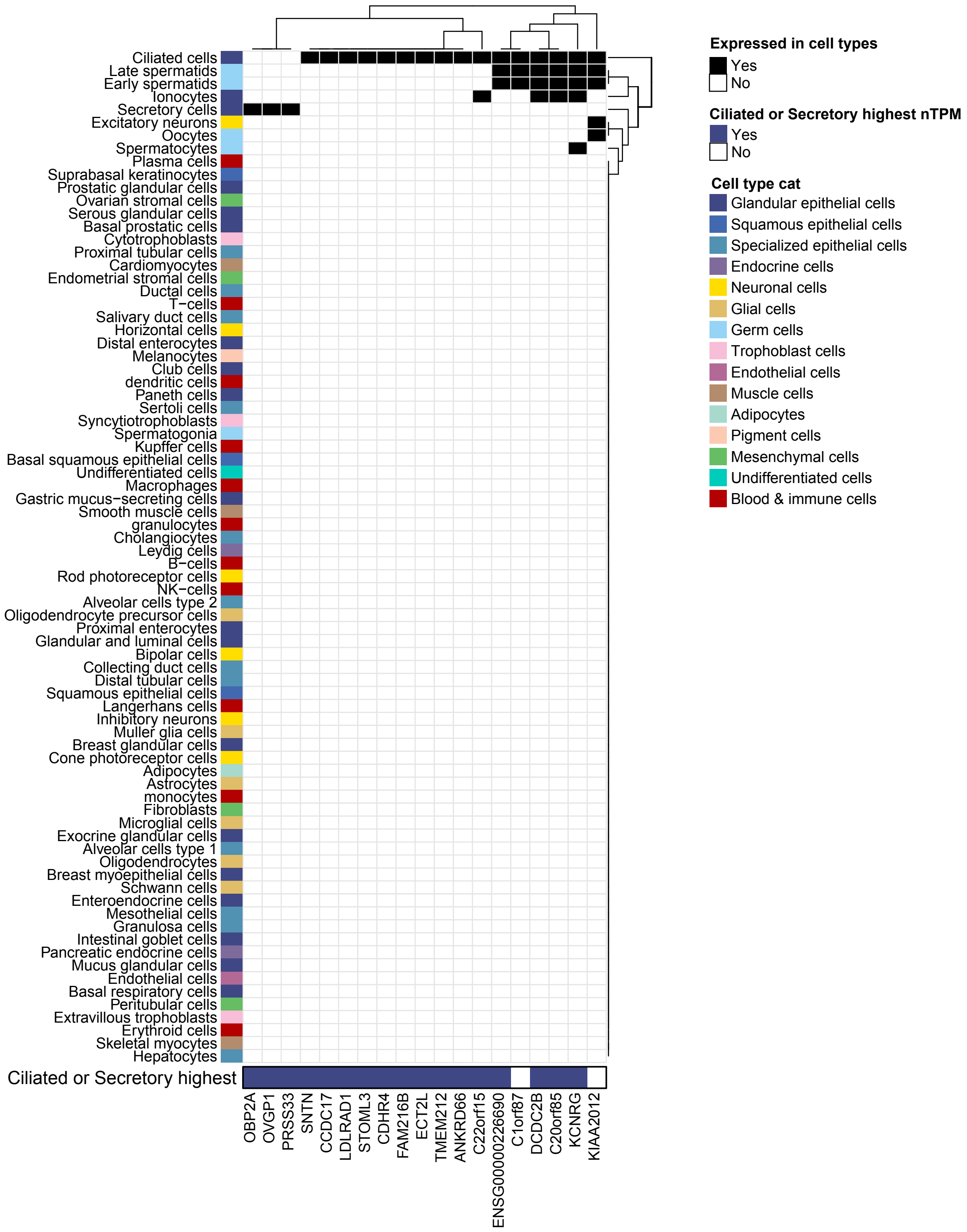

Supplementary Figure 3. Heatmap showing the binary relationship of elevated cell type specificities for 19 genes enriched genes in FT. The bottom legend shows the peak RNA level in Ciliated and/or Secretory cells or not.

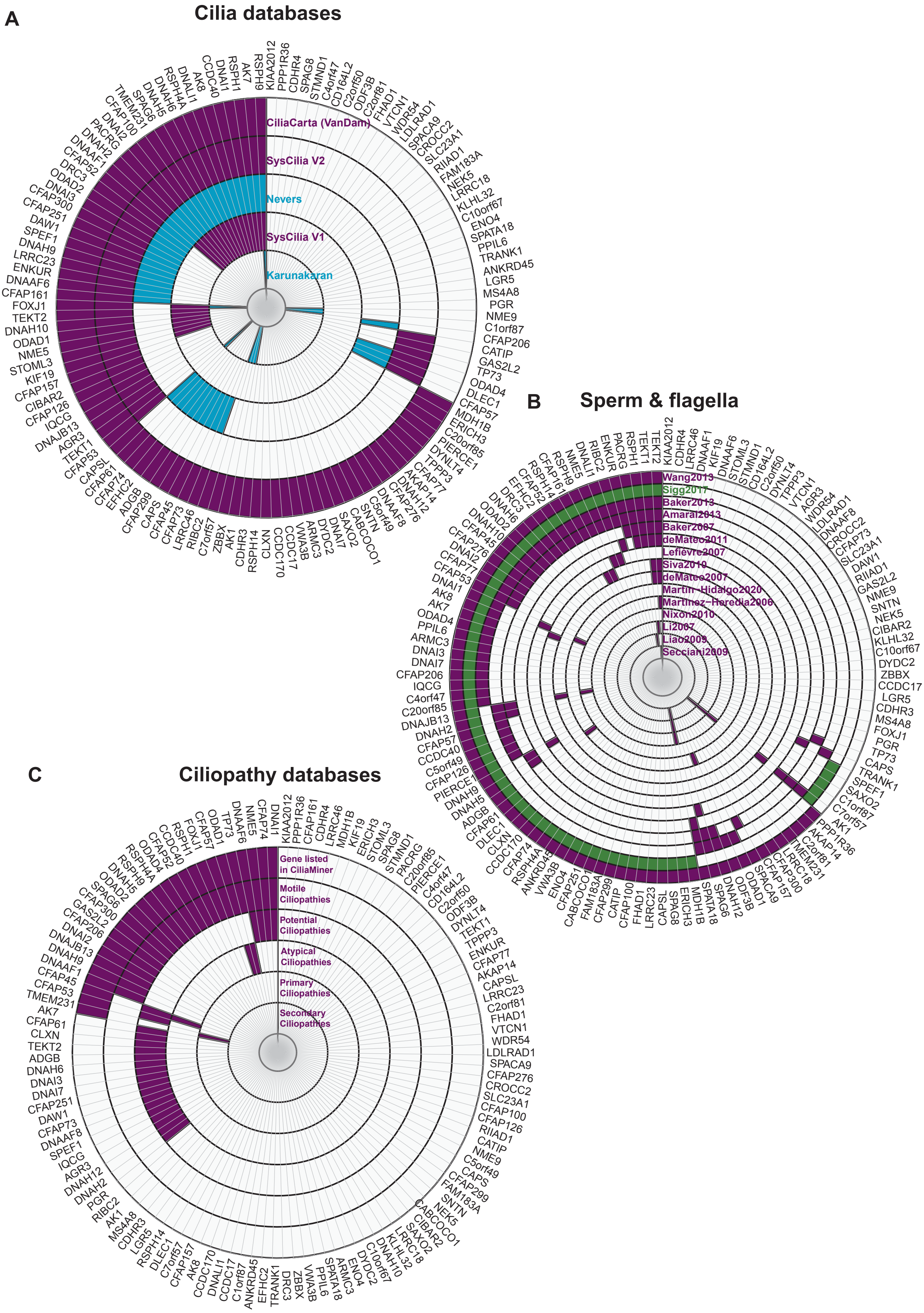

Supplementary Figure 4. Comparing the list of 123 Ciliated FTE cell-specific proteins to proteomics studies on cilia knowledge databases, ciliopathy databases, and Sperm and flagella datasets. The 130 FT proteins examined in IHC in five different databases for cilia-relevant function and expression. The genes/proteins that are present in any or several resources are labeled in purple or blue. The datasets from Nevers & Karunakaran (blue) are based on predictions and phylogenetic approaches, whereas the others in purple are databases based on literature research. Empty bars represent no data in the databases. List of the 123 proteins examined in IHC compared to (A) datasets of flagellar proteins identified in different studies or (B) a database for ciliopathies called “CiliaMiner” (Turan et al., 202324). The proteins that are present in the dataset are filled in purple or green. The dataset shown in green is based on comparing the proteome of sea urchins, sea anemones, and choanoflagellates23; Overlap with this data set provides insight into conserveness of the proteins we identified. The other flagella protein lists all stem from proteomics studies in human sperm and were collected in Amaral et al., 201421. The data set by Martin-Hidalgo et al22 lists proteins with changes in phosphorylation profiles related to sperm motility. The comparison to CiliaMiner was conducted with the data present on CiliaMiner’s webpage (https://kaplanlab.shinyapps.io/ciliaminer/) on December 03, 2022.

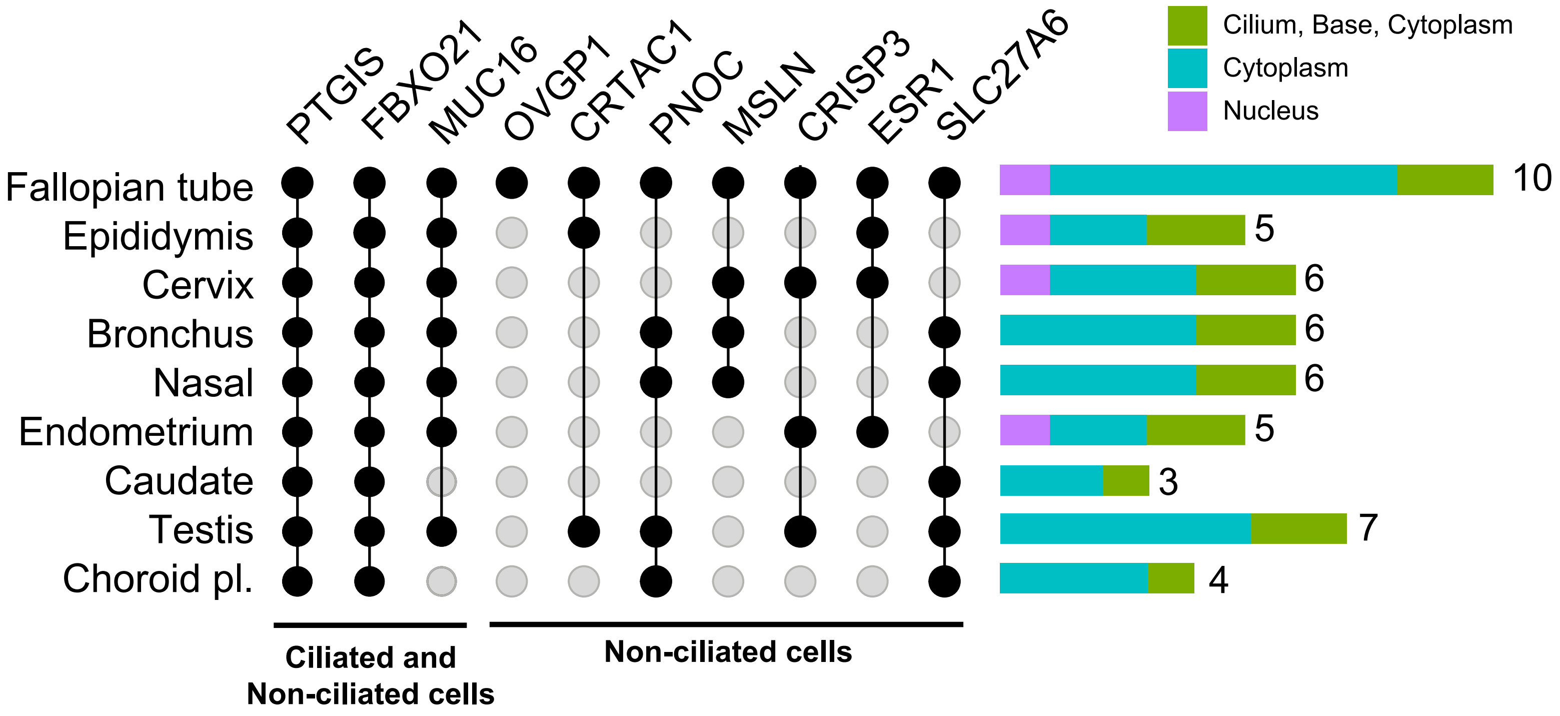

Supplementary Figure 5. The expression of FT-elevated proteins in human tissues with motile ciliated epithelial cells. The highlighted proteins represent those stained in both ciliated and non-ciliated cells, and strictly in non-ciliated cells. Overview dot plot of protein expression with filled circles representing positive staining and light-grey circles representing no staining with the protein names on top. The colored bars and numbers on the right hand side represent a summary of subcellular annotations derived from each tissue and a total count of positive stains in each tissue.

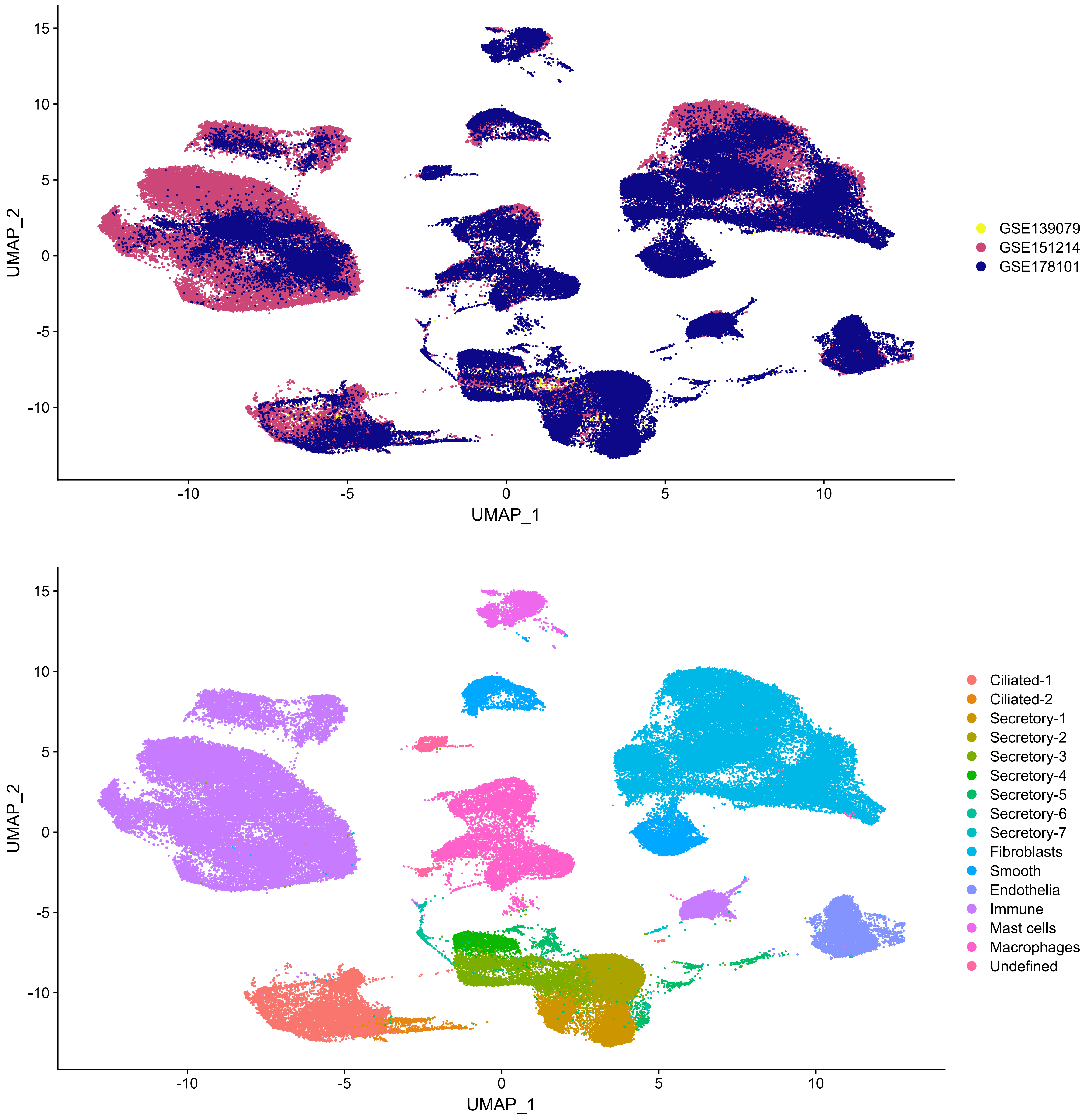

Supplementary Figure 6. Overview of single-cell clusters in human fallopian tube derived from integration of scRNA-seq datasets. An UMAP plot showing the different cell clusters identified in human FT by using integration of three scRNA-seq datasets. Each dot represents a cell. The upper plot highlight cells colored by dataset. The bottom represents the cellular identities assigned after manual annotation (see materials and methods for cell identity markers), comprising of 2 clusters of ciliated FTE cells, 7 secretory FTE cells, fibroblasts, smooth muscle cells, endothelial cells, immune cells (T cells, B cells, NK cells and dendritic cells), mast cells, macrophages. The undefined cell cluster was not used in further the analysis.

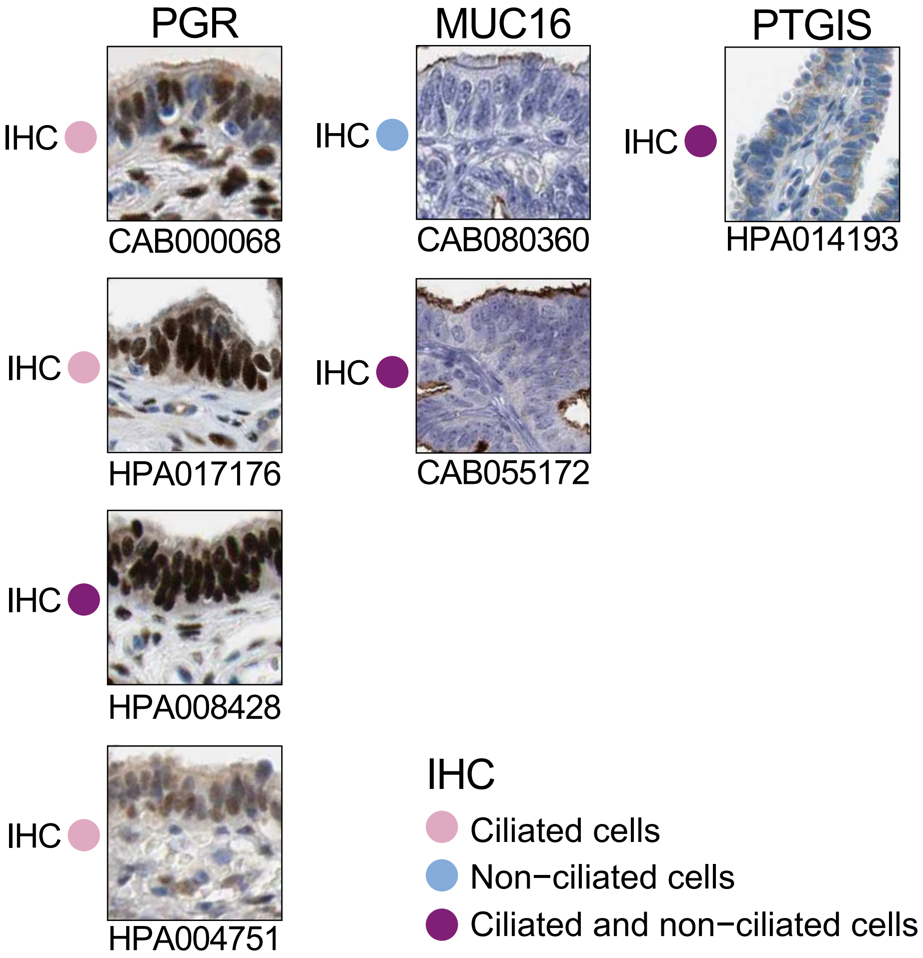

Supplementary Figure 7. IHC stainings in human FT by additional antibodies of proteins included in the study.

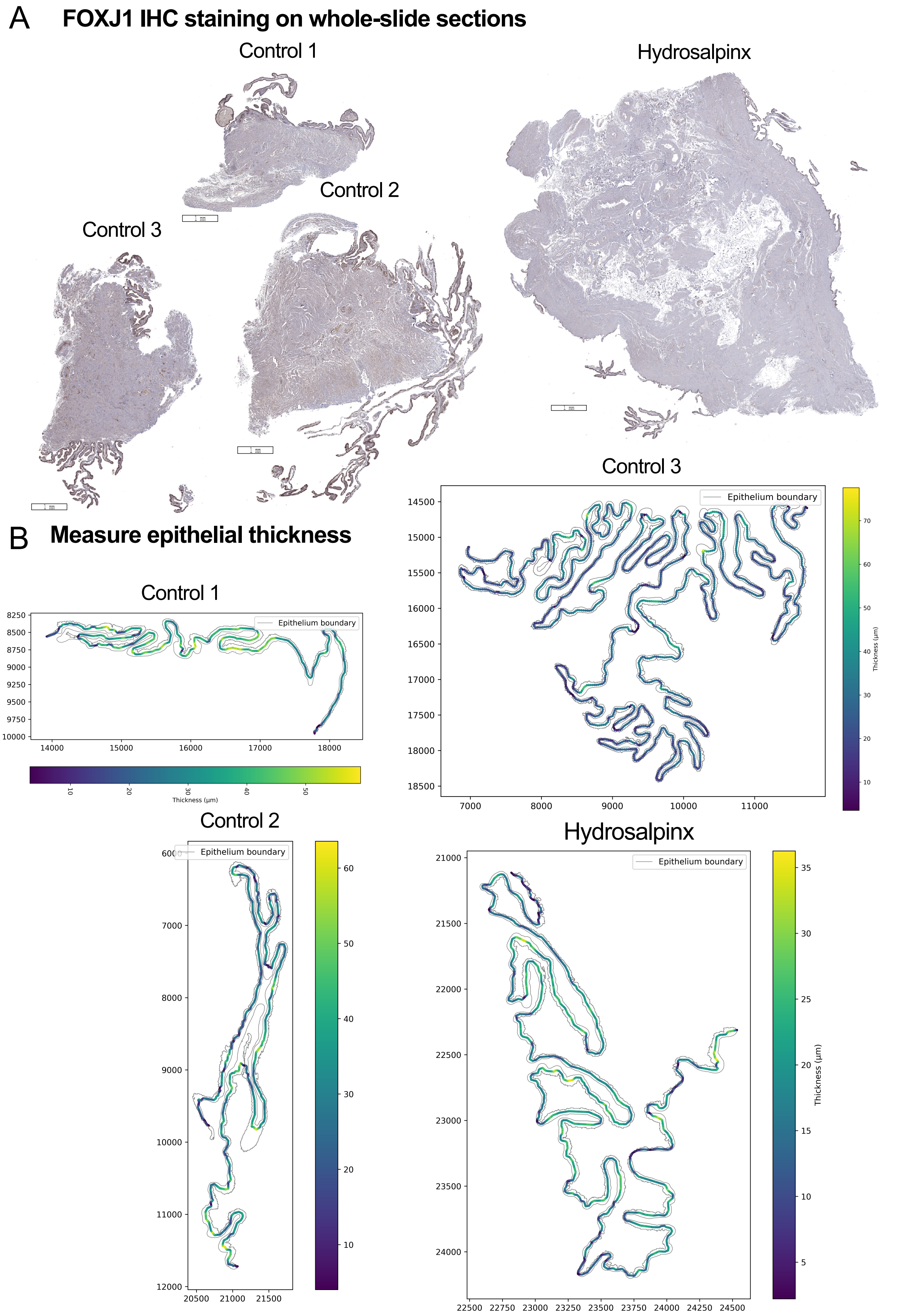

Supplementary Figure 8. A) IHC staining of FOXJ1 in whole-slide hydrosalpinx (HS) and control FT tissues. B) Epithelial width measurement for HS and control samples.

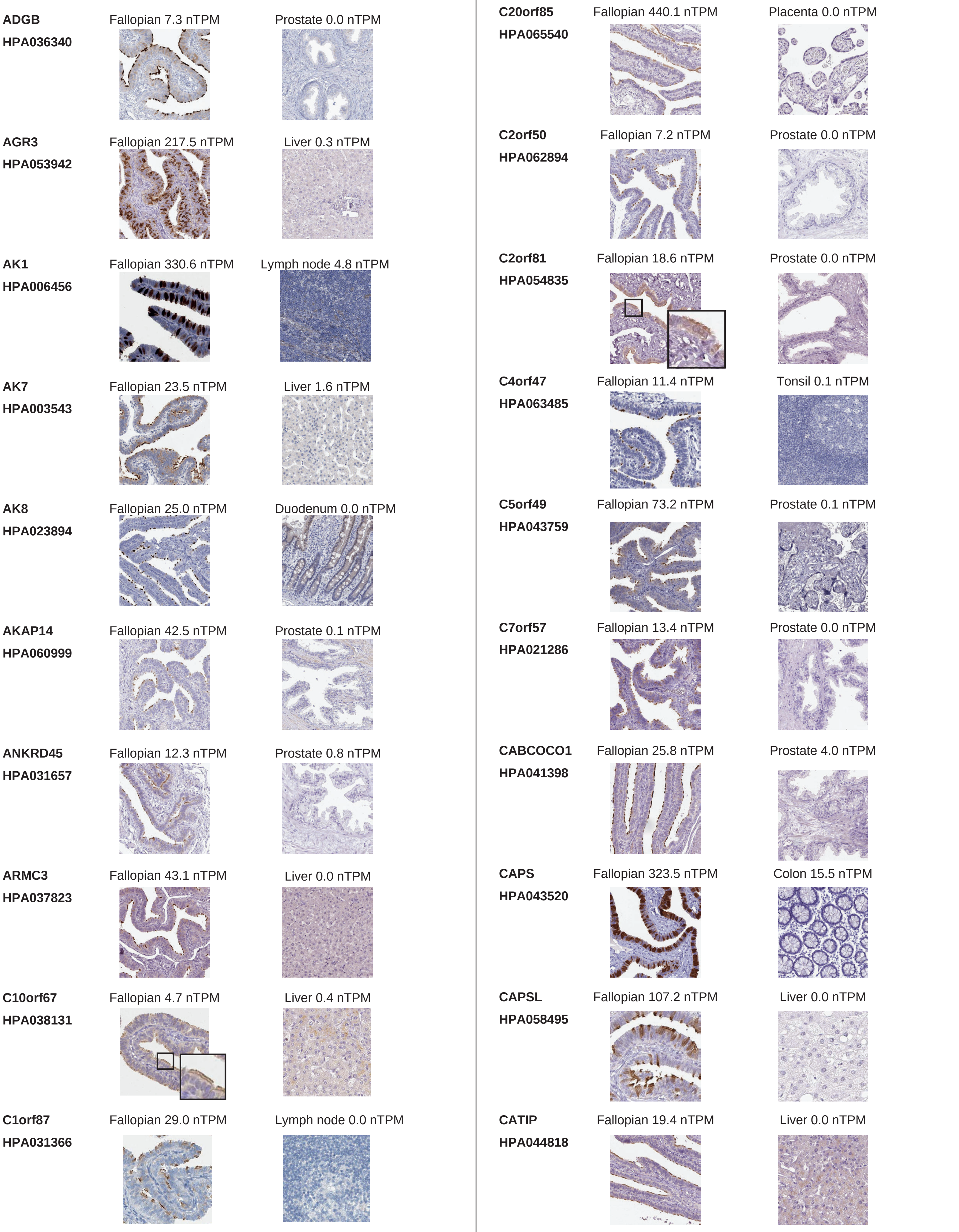
ADGB
HPA036340
Fallopian 7.3 nTPM
Prostate 0.0 nTPM
AGR3
HPA053942
Fallopian 217.5 nTPM
Liver 0.3 nTPM
AK1
HPA006456
Fallopian 330.6 nTPM
Lymph node 4.8 nTPM
AK7
HPA003543
Fallopian 23.5 nTPM
Liver 1.6 nTPM
AK8
HPA023894
Fallopian 25.0 nTPM
Duodenum 0.0 nTPM
AKAP14
HPA060999
Fallopian 42.5 nTPM
Prostate 0.1 nTPM
ANKRD45
HPA031657
Fallopian 12.3 nTPM
Prostate 0.8 nTPM
ARMC3
HPA037823
Fallopian 43.1 nTPM
Liver 0.0 nTPM
C10orf67
HPA038131
Fallopian 4.7 nTPM
Liver 0.4 nTPM
C1orf87
HPA031366
Fallopian 29.0 nTPM
Lymph node 0.0 nTPM
C20orf85
HPA065540
Fallopian 440.1 nTPM
Placenta 0.0 nTPM
C2orf50
HPA062894
Fallopian 7.2 nTPM
Prostate 0.0 nTPM
C2orf81
HPA054835
Fallopian 18.6 nTPM
Prostate 0.0 nTPM
C4orf47
HPA063485
Fallopian 11.4 nTPM
Tonsil 0.1 nTPM
C5orf49
HPA043759
Fallopian 73.2 nTPM
Prostate 0.1 nTPM
C7orf57
HPA021286
Fallopian 13.4 nTPM
Prostate 0.0 nTPM
CABCOCO1
HPA041398
Fallopian 25.8 nTPM
Prostate 4.0 nTPM
CAPS
HPA043520
Fallopian 323.5 nTPM
Colon 15.5 nTPM
CAPSL
HPA058495
Fallopian 107.2 nTPM
Liver 0.0 nTPM
CATIP
HPA044818
Fallopian 19.4 nTPM
Liver 0.0 nTPM

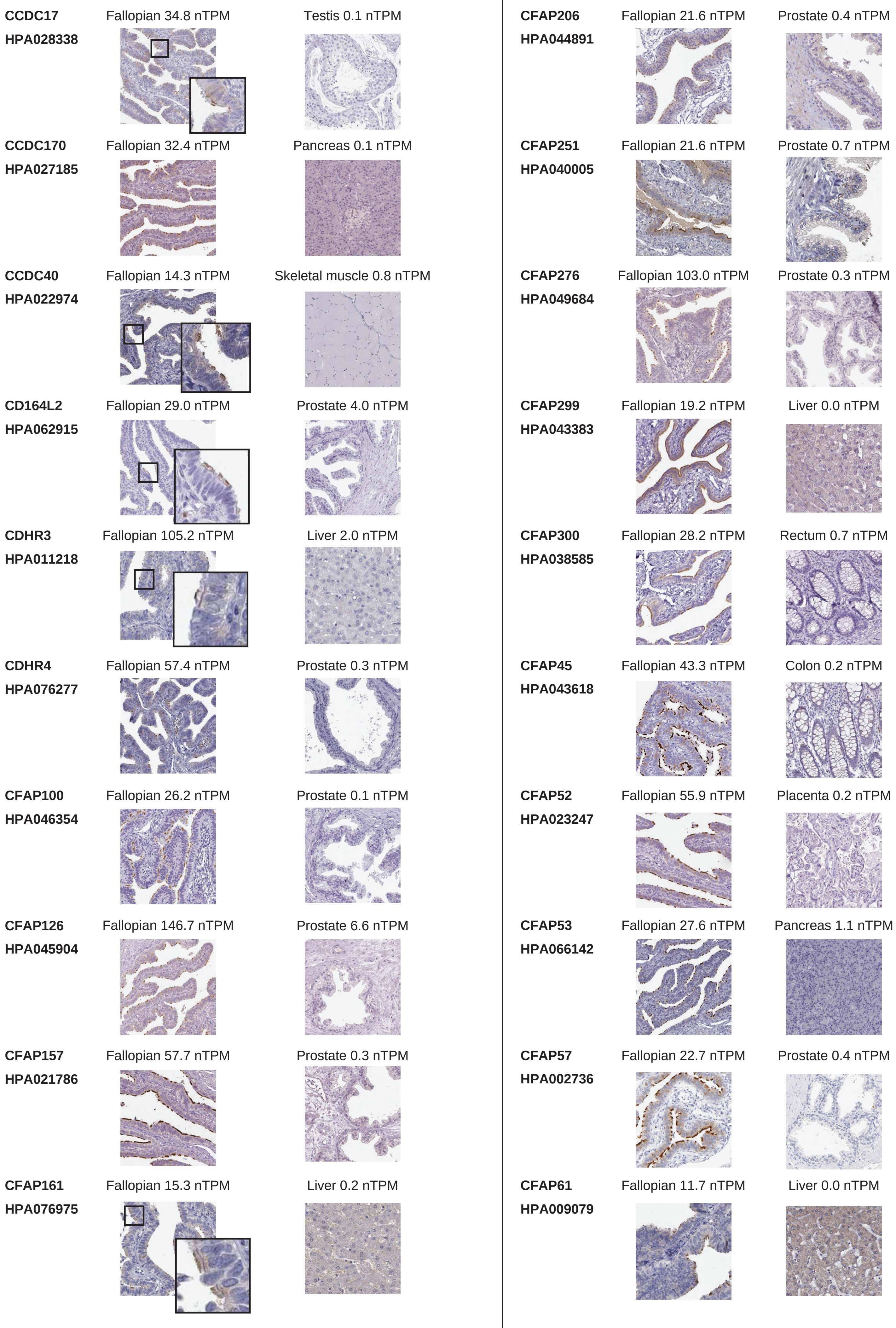
CCDC17
HPA028338
Fallopian 34.8 nTPM
Testis 0.1 nTPM
CCDC170
HPA027185
Fallopian 32.4 nTPM
Pancreas 0.1 nTPM
CCDC40
HPA022974
Fallopian 14.3 nTPM
Skeletal muscle 0.8 nTPM
CD164L2
HPA062915
Fallopian 29.0 nTPM
Prostate 4.0 nTPM
CDHR3
HPA011218
Fallopian 105.2 nTPM
Liver 2.0 nTPM
CDHR4
HPA076277
Fallopian 57.4 nTPM
Prostate 0.3 nTPM
CFAP100
HPA046354
Fallopian 26.2 nTPM
Prostate 0.1 nTPM
CFAP126
HPA045904
Fallopian 146.7 nTPM
Prostate 6.6 nTPM
CFAP157
HPA021786
Fallopian 57.7 nTPM
Prostate 0.3 nTPM
CFAP161
HPA076975
Fallopian 15.3 nTPM
Liver 0.2 nTPM
CFAP206
HPA044891
Fallopian 21.6 nTPM
Prostate 0.4 nTPM
CFAP251
HPA040005
Fallopian 21.6 nTPM
Prostate 0.7 nTPM
CFAP276
HPA049684
Fallopian 103.0 nTPM
Prostate 0.3 nTPM
CFAP299
HPA043383
Fallopian 19.2 nTPM
Liver 0.0 nTPM
CFAP300
HPA038585
Fallopian 28.2 nTPM
Rectum 0.7 nTPM
CFAP45
HPA043618
Fallopian 43.3 nTPM
Colon 0.2 nTPM
CFAP52
HPA023247
Fallopian 55.9 nTPM
Placenta 0.2 nTPM
CFAP53
HPA066142
Fallopian 27.6 nTPM
Pancreas 1.1 nTPM
CFAP57
HPA002736
Fallopian 22.7 nTPM
Prostate 0.4 nTPM
CFAP61
HPA009079
Fallopian 11.7 nTPM
Liver 0.0 nTPM

CFAP73 Fallopian 41.0 nTPM Prostate 0.6 nTPM
HPA048539

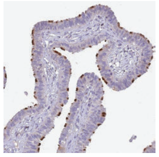
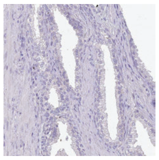

CFAP74 Fallopian 6.7 nTPM Placenta 0.3 nTPM
HPA028521

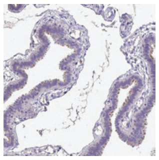
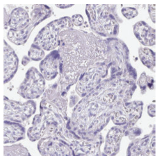

CFAP77 Fallopian 28.7 nTPM Prostate 0.3 nTPM
HPA061069

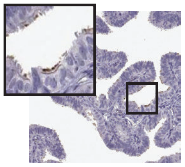
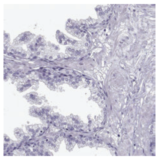

CIBAR2 Fallopian 82.7 nTPM Prostate 0.1 nTPM
HPA041022

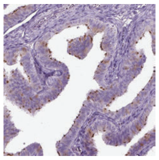
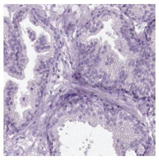

CLXN Fallopian 88.6 nTPM Colon 0.5 nTPM
HPA023527

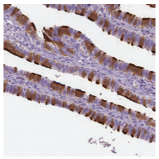
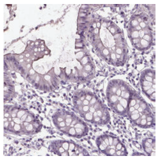

CRISP3 Fallopian 1164.2 nTPM Liver 0.8 nTPM
HPA054392

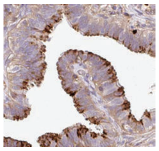
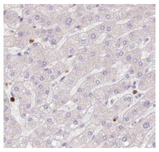

CROCC2 Fallopian 6.8 nTPM Prostate 0.0 nTPM
HPA048678

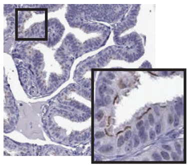
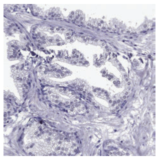

CRTAC1 Fallopian 37.0 nTPM Pancreas 1.3 nTPM
HPA008175

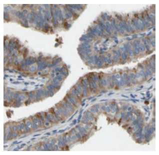
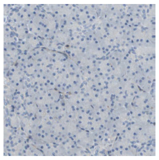

DAW1 Fallopian 31.7 nTPM Liver 0.7 nTPM
HPA046118

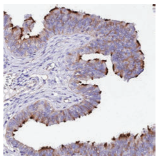
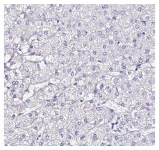

DLEC1 Fallopian 27.1 nTPM Prostate 0.9 nTPM
HPA019077

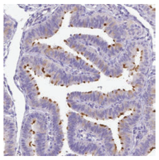
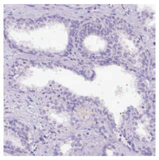

DNAAF1 Fallopian 22.0 nTPM Prostate 0.2 nTPM
HPA074239

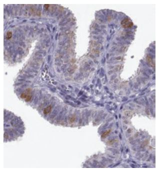
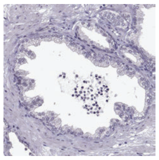

DNAAF6 Fallopian 10.5 nTPM Colon 0.0 nTPM
HPA072496

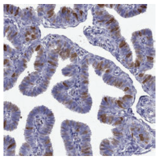
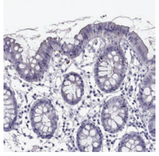

DNAAF8 Fallopian 21.8 nTPM Liver 1.3 nTPM
HPA049468

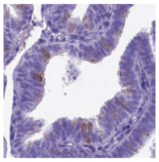
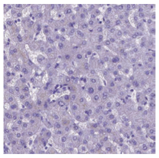

DNAH10 Fallopian 4.0 nTPM Prostate 0.0 nTPM
HPA039066

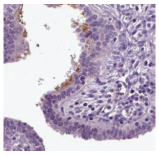
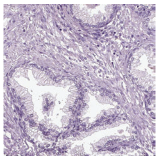

DNAH12 Fallopian 9.9 nTPM Prostate 0.1 nTPM
HPA058203

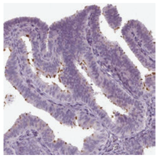
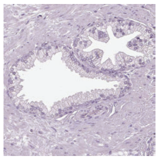

DNAH2 Fallopian 12.6 nTPM Colon 0.2 nTPM
HPA067103

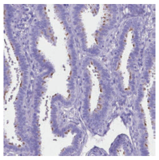
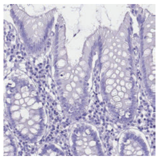

DNAH5 Fallopian 3.4 nTPM Liver 0.9 nTPM
HPA037469

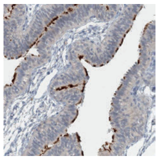
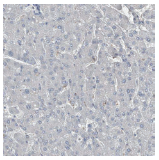

DNAH6 Fallopian 5.3 nTPM Placenta 0.0 nTPM
HPA036391

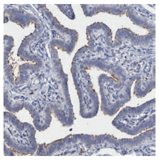
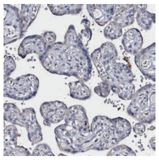

DNAH9 Fallopian 34.1 nTPM Prostate 0.8 nTPM
HPA052641

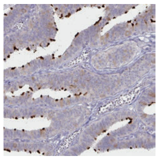
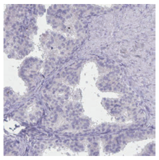

DNAI1 Fallopian 32.8 nTPM Colon 0.0 nTPM
HPA021843

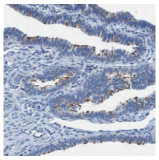
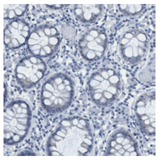

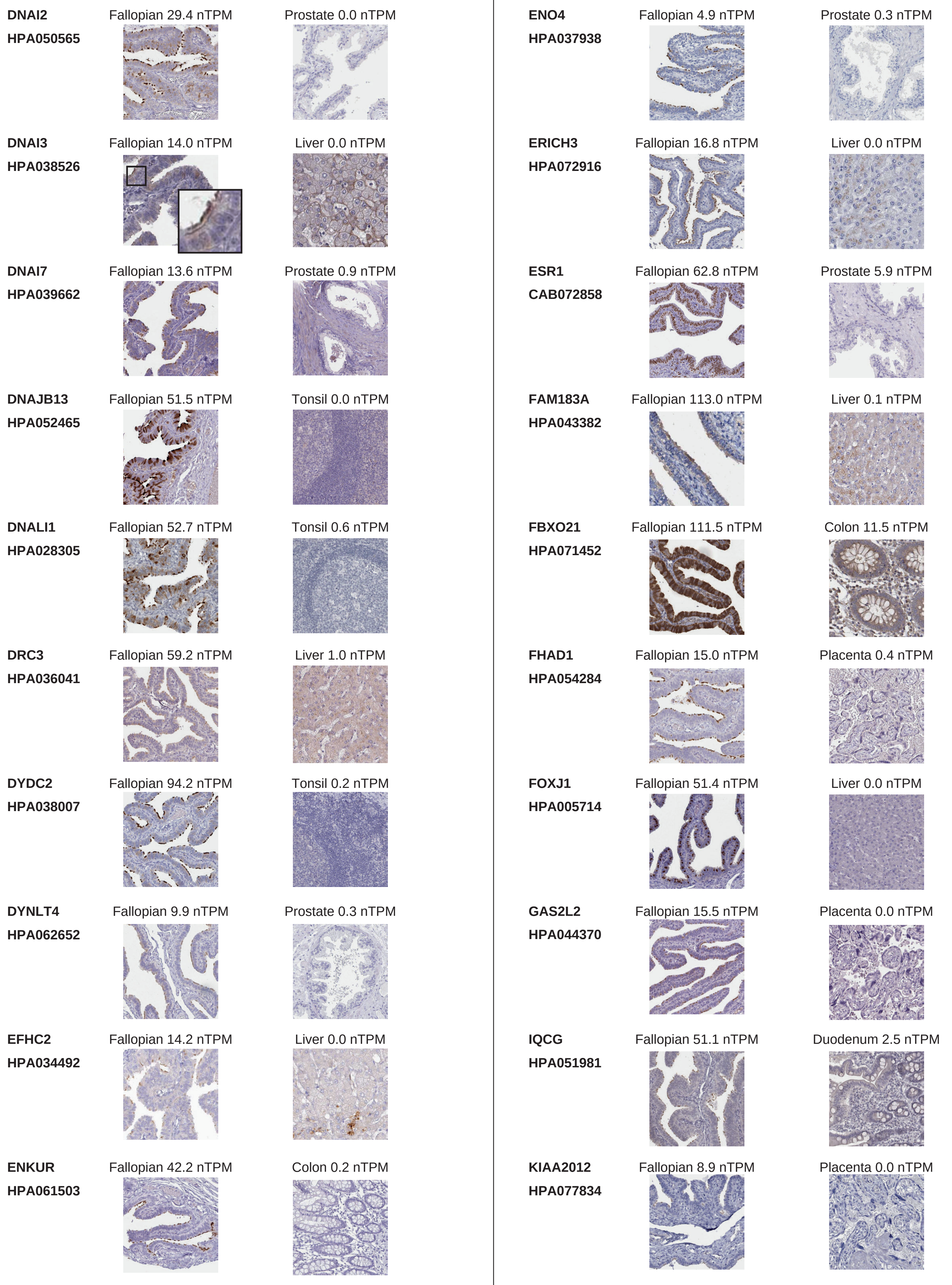
DNAI2
HPA050565
Fallopian 29.4 nTPM
Prostate 0.0 nTPM
DNAI3
HPA038526
Fallopian 14.0 nTPM
Liver 0.0 nTPM
DNAI7
HPA039662
Fallopian 13.6 nTPM
Prostate 0.9 nTPM
DNAJB13
HPA052465
Fallopian 51.5 nTPM
Tonsil 0.0 nTPM
DNALI1
HPA028305
Fallopian 52.7 nTPM
Tonsil 0.6 nTPM
DRC3
HPA036041
Fallopian 59.2 nTPM
Liver 1.0 nTPM
DYDC2
HPA038007
Fallopian 94.2 nTPM
Tonsil 0.2 nTPM
DYNLT4
HPA062652
Fallopian 9.9 nTPM
Prostate 0.3 nTPM
EFHC2
HPA034492
Fallopian 14.2 nTPM
Liver 0.0 nTPM
ENKUR
HPA061503
Fallopian 42.2 nTPM
Colon 0.2 nTPM
ENO4
HPA037938
Fallopian 4.9 nTPM
Prostate 0.3 nTPM
ERICH3
HPA072916
Fallopian 16.8 nTPM
Liver 0.0 nTPM
ESR1
CAB072858
Fallopian 62.8 nTPM
Prostate 5.9 nTPM
FAM183A
HPA043382
Fallopian 113.0 nTPM
Liver 0.1 nTPM
FBXO21
HPA071452
Fallopian 111.5 nTPM
Colon 11.5 nTPM
FHAD1
HPA054284
Fallopian 15.0 nTPM
Placenta 0.4 nTPM
FOXJ1
HPA005714
Fallopian 51.4 nTPM
Liver 0.0 nTPM
GAS2L2
HPA044370
Fallopian 15.5 nTPM
Placenta 0.0 nTPM
IQCG
HPA051981
Fallopian 51.1 nTPM
Duodenum 2.5 nTPM
KIAA2012
HPA077834
Fallopian 8.9 nTPM
Placenta 0.0 nTPM

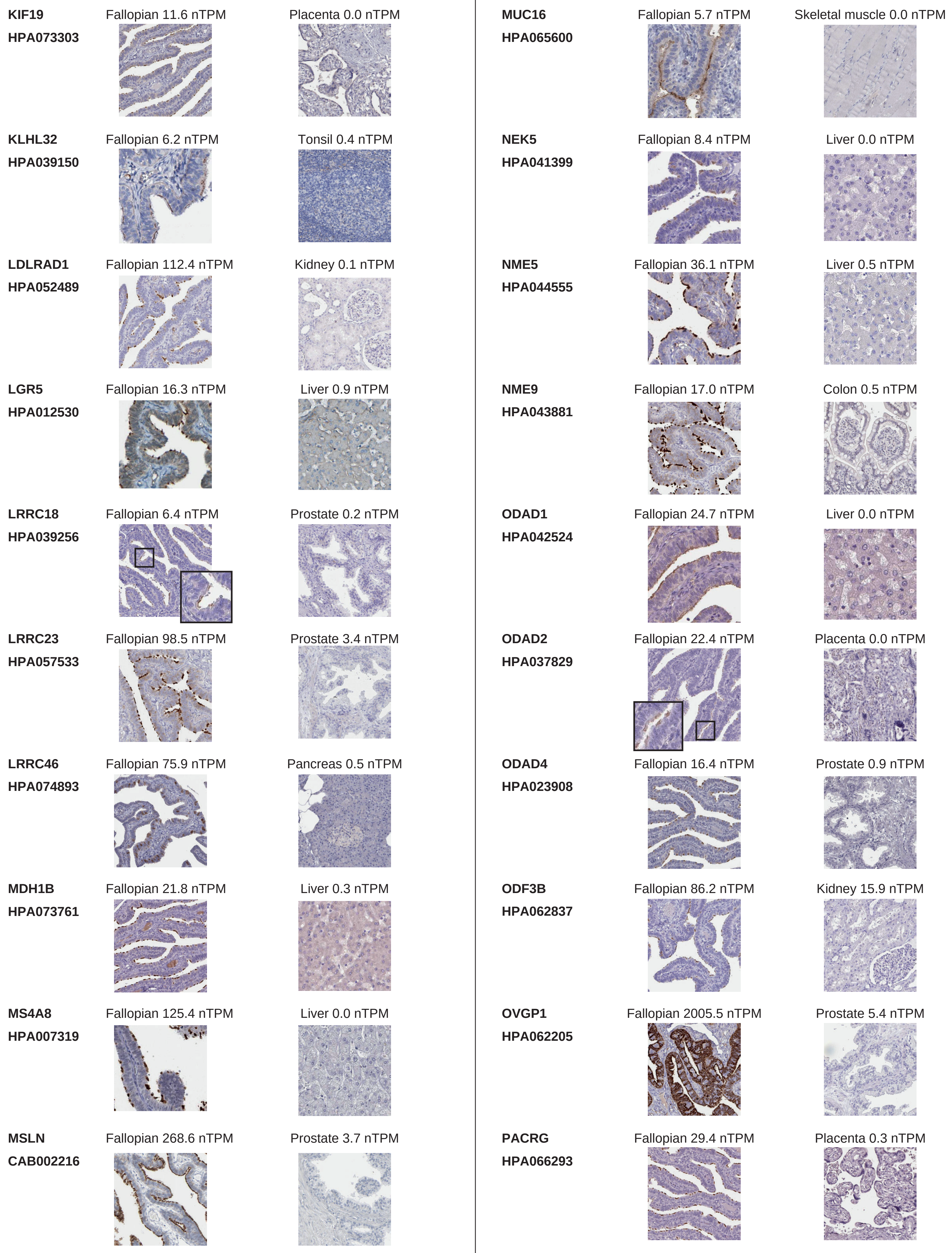
KIF19
HPA073303
Fallopian 11.6 nTPM
Placenta 0.0 nTPM
KLHL32
HPA039150
Fallopian 6.2 nTPM
Tonsil 0.4 nTPM
LDLRAD1
HPA052489
Fallopian 112.4 nTPM
Kidney 0.1 nTPM
LGR5
HPA012530
Fallopian 16.3 nTPM
Liver 0.9 nTPM
LRRC18
HPA039256
Fallopian 6.4 nTPM
Prostate 0.2 nTPM
LRRC23
HPA057533
Fallopian 98.5 nTPM
Prostate 3.4 nTPM
LRRC46
HPA074893
Fallopian 75.9 nTPM
Pancreas 0.5 nTPM
MDH1B
HPA073761
Fallopian 21.8 nTPM
Liver 0.3 nTPM
MS4A8
HPA007319
Fallopian 125.4 nTPM
Liver 0.0 nTPM
MSLN
CAB002216
Fallopian 268.6 nTPM
Prostate 3.7 nTPM
MUC16
HPA065600
Fallopian 5.7 nTPM
Skeletal muscle 0.0 nTPM
NEK5
HPA041399
Fallopian 8.4 nTPM
Liver 0.0 nTPM
NME5
HPA044555
Fallopian 36.1 nTPM
Liver 0.5 nTPM
NME9
HPA043881
Fallopian 17.0 nTPM
Colon 0.5 nTPM
ODAD1
HPA042524
Fallopian 24.7 nTPM
Liver 0.0 nTPM
ODAD2
HPA037829
Fallopian 22.4 nTPM
Placenta 0.0 nTPM
ODAD4
HPA023908
Fallopian 16.4 nTPM
Prostate 0.9 nTPM
ODF3B
HPA062837
Fallopian 86.2 nTPM
Kidney 15.9 nTPM
OVGP1
HPA062205
Fallopian 2005.5 nTPM
Prostate 5.4 nTPM
PACRG
HPA066293
Fallopian 29.4 nTPM
Placenta 0.3 nTPM

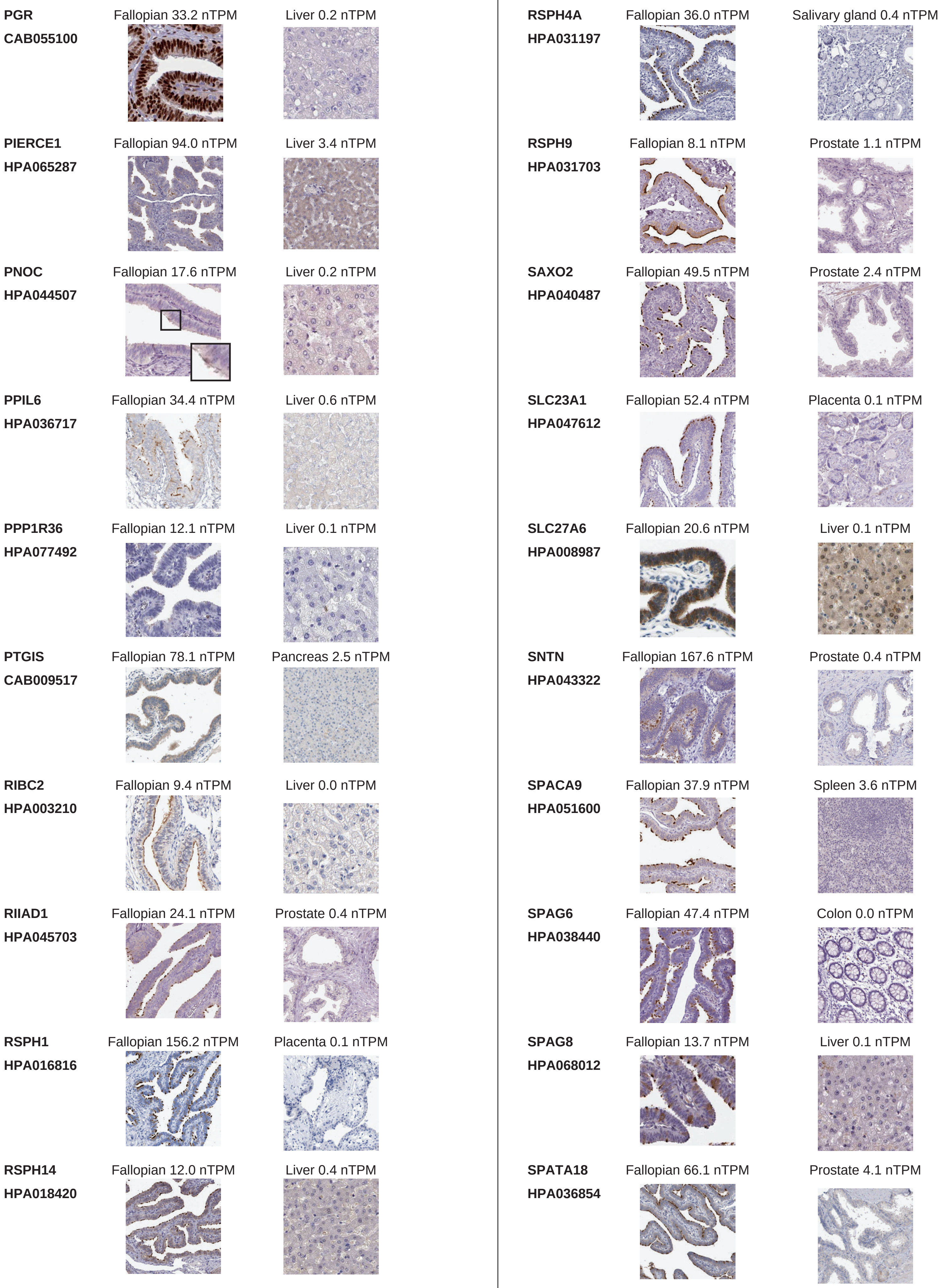
PGR
CAB055100
Fallopian 33.2 nTPM
Liver 0.2 nTPM
PIERCE1
HPA065287
Fallopian 94.0 nTPM
Liver 3.4 nTPM
PNOC
HPA044507
Fallopian 17.6 nTPM
Liver 0.2 nTPM
PPIL6
HPA036717
Fallopian 34.4 nTPM
Liver 0.6 nTPM
PPP1R36
HPA077492
Fallopian 12.1 nTPM
Liver 0.1 nTPM
PTGIS
CAB009517
Fallopian 78.1 nTPM
Pancreas 2.5 nTPM
RIBC2
HPA003210
Fallopian 9.4 nTPM
Liver 0.0 nTPM
RIIAD1
HPA045703
Fallopian 24.1 nTPM
Prostate 0.4 nTPM
RSPH1
HPA016816
Fallopian 156.2 nTPM
Placenta 0.1 nTPM
RSPH14
HPA018420
Fallopian 12.0 nTPM
Liver 0.4 nTPM
RSPH4A
HPA031197
Fallopian 36.0 nTPM
Salivary gland 0.4 nTPM
RSPH9
HPA031703
Fallopian 8.1 nTPM
Prostate 1.1 nTPM
SAXO2
HPA040487
Fallopian 49.5 nTPM
Prostate 2.4 nTPM
SLC23A1
HPA047612
Fallopian 52.4 nTPM
Placenta 0.1 nTPM
SLC27A6
HPA008987
Fallopian 20.6 nTPM
Liver 0.1 nTPM
SNTN
HPA043322
Fallopian 167.6 nTPM
Prostate 0.4 nTPM
SPACA9
HPA051600
Fallopian 37.9 nTPM
Spleen 3.6 nTPM
SPAG6
HPA038440
Fallopian 47.4 nTPM
Colon 0.0 nTPM
SPAG8
HPA068012
Fallopian 13.7 nTPM
Liver 0.1 nTPM
SPATA18
HPA036854
Fallopian 66.1 nTPM
Prostate 4.1 nTPM

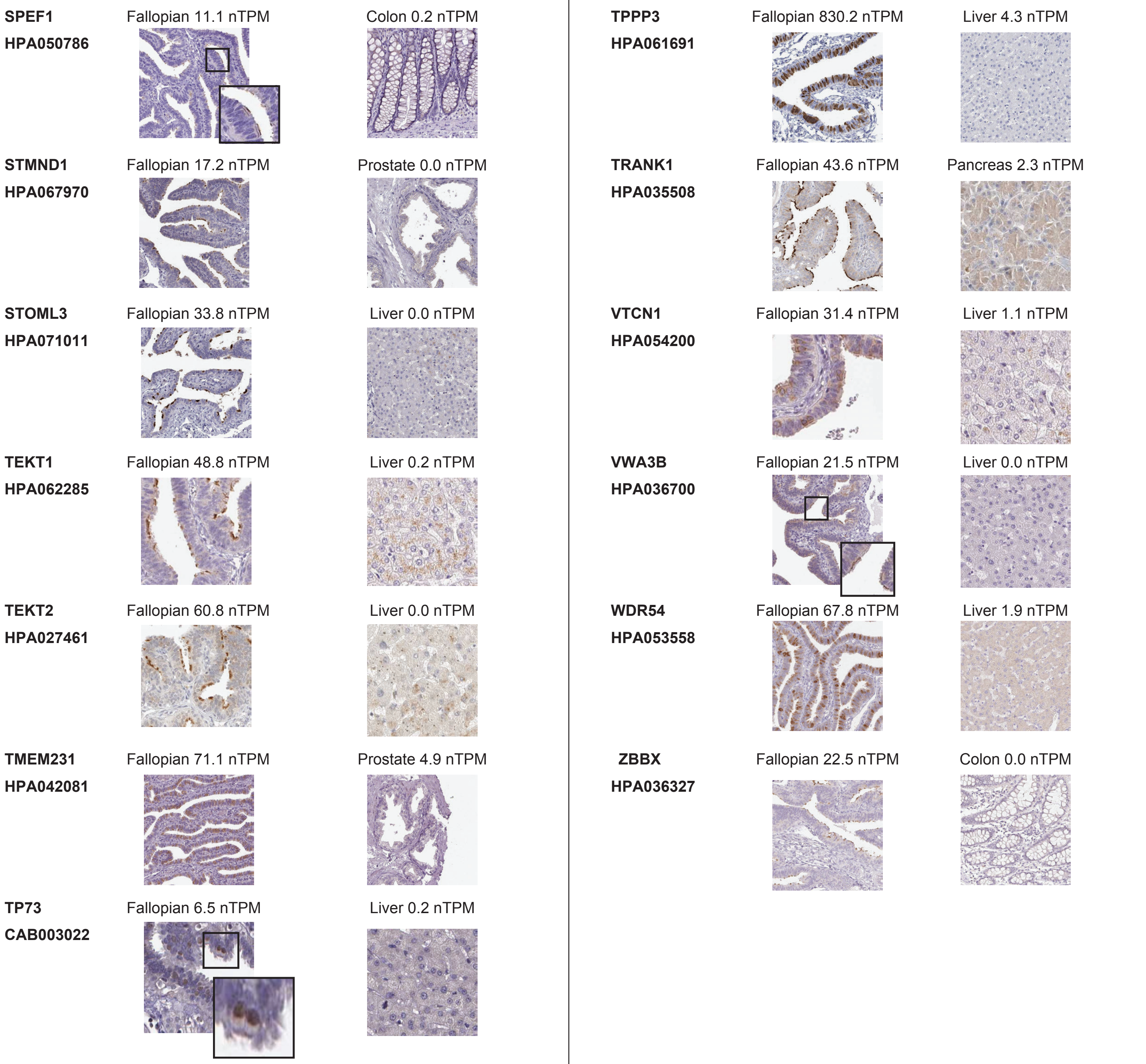

Supplementary Figure 9. Validation of antibody specificity using positive (FT tissue) and negative control stainings. Negative control tissues were selected based on low mRNA expression, or below detection threshold, and were expected to lack or exhibit very low abundance of the target protein.
