# Supplementary material for: A high-resolution spatial map of cilia-associated proteins in the human fallopian tube
Source: Nat Commun. 2026 Apr 20;17:3616. doi: 10.1038/s41467-026-71692-6 (PMC13096173; doi:10.1038/s41467-026-71692-6)
Supplement: Supplementary file 2 — Description of Additional Supplementary Files [file 41467_2026_71692_MOESM2_ESM.pdf]

Description of Additional Supplementary Files

File Name: Supplementary Data 1

Description: Overview of RNA specificity and distribution for all FT-elevated genes. Detection cut-off <1 nTPM. For distribution scores: detected in fallopian tube only = only fallopian tube tissue above detection cut-off, detected in some = in more than one but less than one third of tissues, detected in many = in at least a third but not all tissues, detected in all= above detection cut-off in all tissues. For RNA specifity: tissue enriched = nTPM in a particular tissue at least four times any other tissue, group enriched = nTPM levels in a group of 2-5 tissues at least four times any other tissue, tissue enhanced = nTPM levels in one or several (1-5 tissues) at least four times the mean value of all other tissues..

File Name: Supplementary Data 2

Description: RNA specificity categories and distribution categories as well as expression levels (nTPM) for genes elevated in Fallopian tube in all tissues and cells available in the HPA.

File Name: Supplementary Data 3

Description: GO enrichment statistical analysis of elevated genes in fallopian tube. Significance was determined using Benjamini-Hochberg adjusted p-values to control for multiple testing.

File Name: Supplementary Data 4

Description: General gene and protein data reliability of all FT elevated genes. The data was downloaded from the HPA and Uniprot.org.

File Name: Supplementary Data 5

Description: Manual annotation data.

File Name: Supplementary Data 6

Description: Clinical data of additional control (C1-3) and hydrosalpinx (HS) samples.

File Name: Supplementary Data 7

Description: All antibodies used in the IHC profiling.

File Name: Supplementary Data 8

Description: Differential abundance for protein groups in $FOXJ1^{+}$ versus $FOXJ1^{-}$ cells based on Deep Visual Proteomics. The statistical test applied is a two-talied Student's T-test.
