# Supplementary material for: A high-resolution spatial map of cilia-associated proteins in the human fallopian tube
Source: Nat Commun. 2026 Apr 20;17:3616. doi: 10.1038/s41467-026-71692-6 (PMC13096173; doi:10.1038/s41467-026-71692-6)
Supplement: Supplementary file 13 — Source Data [file 41467_2026_71692_MOESM13_ESM.zip › Source_Data/Source_data_Figure_4_5B_ 6B_7A_7B_7D_Supplementary_Figure_7_8A_9.pdf]

Representative immunohistochemical images shown in Figures 4, 5B, 6B, 7A, 7B and 7D, as well as Supplementary Figures 7, 8A and 9, are available in the Human Protein Atlas (HPA; v25.proteinatlas.org) and can be accessed by gene name in the Tissue Resource. These images are also available via BioStudies under accession code S-BIAD1031.
