# Supplementary material for: A high-resolution spatial map of cilia-associated proteins in the human fallopian tube
Source: Nat Commun. 2026 Apr 20;17:3616. doi: 10.1038/s41467-026-71692-6 (PMC13096173; doi:10.1038/s41467-026-71692-6)
Supplement: Supplementary file 13 — Source Data [file 41467_2026_71692_MOESM13_ESM.zip › Source_Data/Source_data_Supplementary_Figure_6.pdf]

The single-cell RNA-seq data used to generate the UMAP representations shown in Supplementary Figure 6 are available in the Gene Expression Omnibus (GEO) under accession codes GSE139079, GSE151214, and GSE178101. UMAP visualizations were generated from an integrated dataset (rPCA-based integration) using Seurat; the full analysis pipeline is available at https://github.com/LindskogLab/Spatial-map-of-cilia-associated-proteins-in-the-human-fallopian-tube
